# Pretreatment level of serum sialic acid predicts both qualitative and quantitative bone metastases of prostate cancer

**DOI:** 10.3389/fendo.2024.1338420

**Published:** 2024-02-07

**Authors:** Jingtao Sun, Tian Tian, Naiqiang Wang, Xuehui Jing, Laiyuan Qiu, Haochen Cui, Zhao Liu, Jikai Liu, Lei Yan, Dawei Li

**Affiliations:** ^1^ Department of Urology, Qilu Hospital of Shandong University, Jinan, China; ^2^ Respiratory and Critical Care Medicine Department, Qilu Hospital of Shandong University, Jinan, China; ^3^ Department of Urology, Yucheng People’s Hospital, Dezhou, China

**Keywords:** serum sialic acid, bone metastases, prostate cancer, low-volume disease, high-volume disease

## Abstract

**Background:**

Recently, serum sialic acid (SA) has emerged as a distinct prognostic marker for prostate cancer (PCa) and bone metastases, warranting differential treatment and prognosis for low-volume (LVD) and high-volume disease (HVD). In clinical settings, evaluating bone metastases can prove advantageous.

**Objectives:**

We aimed to establish the correlation between SA and both bone metastasis and HVD in newly diagnosed PCa patients.

**Methods:**

We conducted a retrospective analysis of 1202 patients who received a new diagnosis of PCa between November 2014 and February 2021. We compared pretreatment SA levels across multiple groups and investigated the associations between SA levels and the clinical parameters of patients. Additionally, we compared the differences between HVD and LVD. We utilized several statistical methods, including the non-parametric Mann-Whitney U test, Spearman correlation, receiver operating characteristic (ROC) curve analysis, and logistic regression.

**Results:**

The results indicate that SA may serve as a predictor of bone metastasis in patients with HVD. ROC curve analysis revealed a cut-off value of 56.15 mg/dL with an area under the curve of 0.767 (95% CI: 0.703-0.832, *P* < 0.001) for bone metastasis versus without bone metastasis and a cut-off value of 65.80 mg/dL with an area under the curve of 0.766 (95% CI: 0.644-0.888, *P* = 0.003) for HVD versus LVD. Notably, PCa patients with bone metastases exhibited significantly higher SA levels than those without bone metastases, and HVD patients had higher SA levels than LVD patients. In comparison to the non-metastatic and LVD cohorts, the cohort with HVD exhibited higher levels of alkaline phosphatase (AKP) (median, 122.00 U/L), fibrinogen (FIB) (median, 3.63 g/L), and prostate-specific antigen (PSA) (median, 215.70 ng/mL), as well as higher Gleason scores (> 7). Multivariate logistic regression analysis demonstrated that an SA level of > 56.15 mg/dL was independently associated with the presence of bone metastases in PCa patients (OR = 2.966, *P* = 0.018), while an SA level of > 65.80 mg/dL was independently associated with HVD (OR = 1.194, *P* = 0.048).

**Conclusion:**

The pretreatment serum SA level is positively correlated with the presence of bone metastases.

## 1 Introduction

Prostate cancer (PCa) is a prevalent malignancy that originates from the male reproductive system. Despite a lower incidence rate among Asians compared to Americans and Europeans (1), the mortality and morbidity rates associated with PCa are on the rise in China due to improvements in living standards, lifestyle changes, and advancements in screening methods (2, 3). The use of prostate-specific antigen (PSA) for population screening, diagnosis, and monitoring of PCa has been a subject of controversy since its purification (4). Although PSA screening has some benefits, such as early detection of PCa, it also has several drawbacks, such as high costs, long waiting times, limited sensitivity, and low specificity. Current evidence suggests that the overall public health impact of PSA screening is not significantly greater than its benefits (5–7). Various treatment options are available for patients diagnosed with early-stage PCa, including surgical removal, chemotherapy, and castration therapy (8). Metastasis is the leading cause of death in PCa, with lymph nodes near the primary tumor being the initial sites of metastasis, followed by bone metastases (9). Currently, there exists no efficacious treatment for patients afflicted with bone metastases (10, 11). Multiple studies have demonstrated that individuals with bone metastases stemming from PCa have a poorer prognosis and a reduced quality of life (12). Consequently, the timely identification of bone metastases in PCa patients is of paramount importance. Although a bone scan is frequently employed for early detection, its specificity is limited, resulting in a high incidence of false-positive results. Given a thorough comprehension of PCa, emission computerized tomography (ECT) is regarded as one of the most effective techniques for detecting bone metastases in patients at present (13–15). Despite the expenses and radioactivity associated with the procedure, it is deemed a valuable investment. Thus, there is an urgent need for novel biomarkers to aid in the identification and long-term monitoring of bone metastases in PCa patients.

Acetylneuraminic acid (sialic acid [SA]), a nine-carbon monosaccharide initially reported in 1957, may serve as a potential candidate. The termination of glycoprotein side chains and glycolipid side chains, which are fundamental constituents of cellular membranes, occurs at this location (16). Furthermore, this agent not only provides cytoprotection but also safeguards cell membranes (17). Multiple studies have established a correlation between elevated levels of SA and cancer, as evidenced in patients (18) with ovarian cancer (19), breast cancer (20), oral cancer (21), and neck cancer (22). Additionally, certain chemical agents have been shown to enhance disease invasion by altering SA levels or impeding SA production, as demonstrated in some studies (23, 24). A noteworthy correlation has been established between SA levels and PCa (25, 26). Notably, patients with PCa who exhibit elevated SA levels are more prone to exhibit heightened levels of PSA, lactate dehydrogenase (LDH), and α-L-fucosidase (AFU), a higher Gleason score, and a higher metastasis incidence rate (26). At present, the primary treatment modalities for PCa include androgen deprivation therapy, radiation therapy, ablative therapy, chemotherapy, and emerging immunotherapies. In clinical settings, it may be necessary to employ multiple approaches simultaneously (27–29). However, this does not diminish the significance of thoroughly assessing the metastatic load of patients prior to treatment in order to determine appropriate treatment options and prognoses (30, 31). Currently, investigations into the correlation between pretreatment SA levels and the metastatic burden of PCa are limited. Therefore, our study was designed to examine this potential association through retrospective data analysis.

## 2 Materials and methods

### 2.1 Patients selection

**Figure 1:**
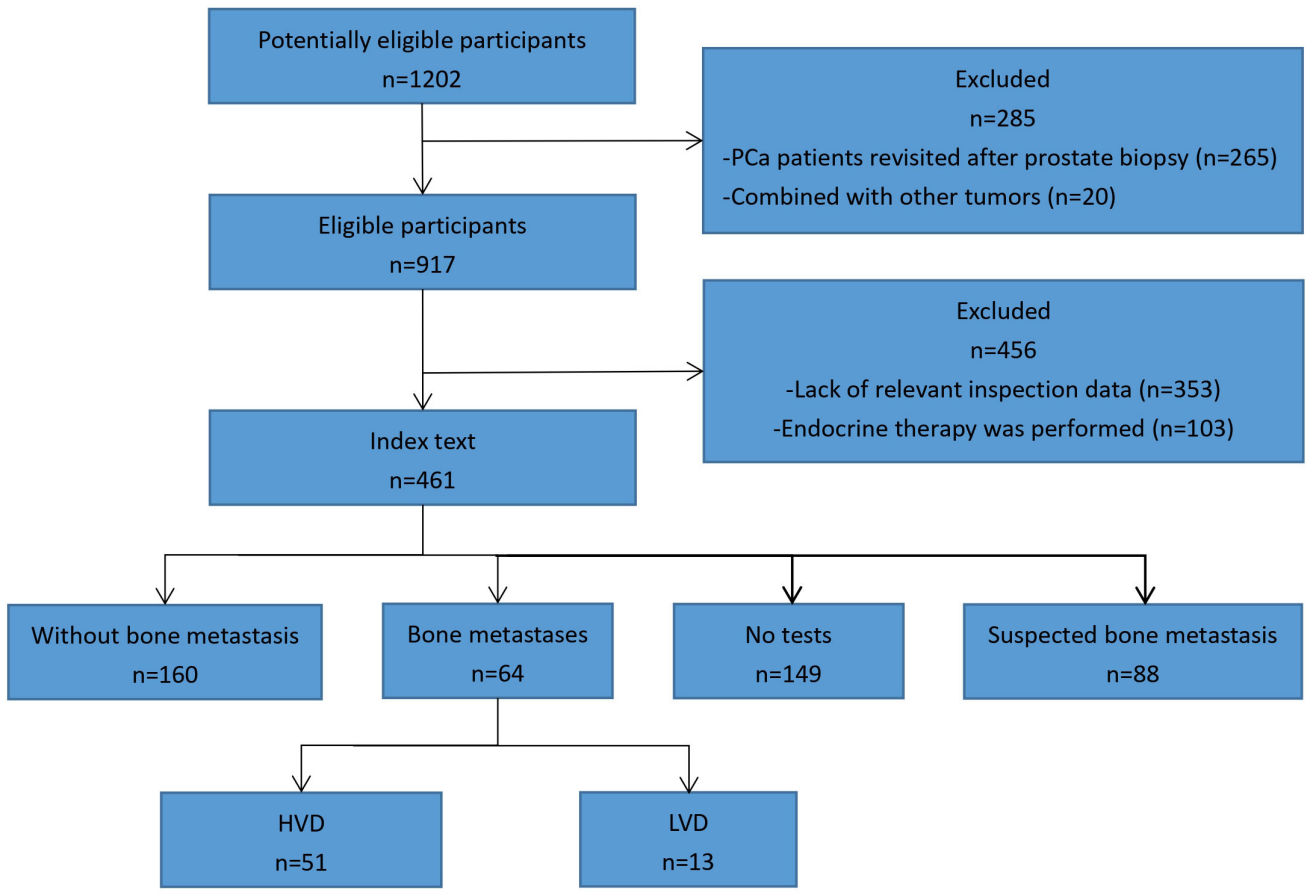
The data screening process of the study.

This retrospective analysis was performed on 1202 PCa patients diagnosed by prostate biopsy in our hospital’s Urology Department between January 2014 and January 2021. The inclusion criteria encompassed the following: (I) Absence of any prior cancer diagnosis; (II) Absence of hematologic disorders to prevent potential confounding of hematologic markers; (III) Availability of comprehensive postoperative pathology findings; (IV) Availability of comprehensive clinical data; (V) Diagnosed with PCa. Our study excluded patients with any one or more of the following conditions: (I) Patients with hematological diseases (Individuals diagnosed with hematological disorders, or prescribed medications known to potentially alter blood markers), known infections, and other malignancies; (II) Patients who had undergone prostate surgery (such as transurethral resection) before their biopsies; (III) Pathologically diagnosed patients with prostatic intraepithelial neoplasms and atypical small acinar proliferations; (IV) Patients with incomplete clinical data. Following that, we collected the following information from the medical records of eligible patients: age at the time of diagnosis, AKP, LDH, AFU, FIB, and PSA levels, pathological characteristics (including pathological grade and stage), and ECT results. The data screening process of the study is shown in **Figure 1**. Our study was approved by the Medical Ethics Committee of Qilu Hospital of Shandong University (KYLL-202208-044-1).

### 2.2 Data collection

Data were obtained from the database of Qilu Hospital of Shangdong University, including age at the time of diagnosis, AKP, LDH, AFU, FIB, and PSA levels, pathological characteristics (including pathological grade and stage), and ECT results. Pathological grades were evaluated using the Gleason system and pathological stages were evaluated according to TNM 2021. Assessment of bone metastasis was performed using ECT, where a positive test result indicated the presence of metastatic lesions. When an isolated condensed radionuclide spot was observed, this was considered as one metastatic lesion.

### 2.3 Measurement of hematology indices

Five milliliters of venous blood was drawn from each patient after they had fasted for 12 h in the early morning before any clinical intervention was given. During the experiment, test tubes containing a clot activator and gel were used to store blood samples, which coagulated naturally at room temperature. The samples were centrifuged for 10 min at 2000 rpm. After the serum was obtained, the AKP, LDH, AFU, and SA concentrations were determined using a Roche Cobas 8000 automatic analyzer. The normal concentration range for AKP was 45.00 to 125.00 U/L; the normal concentration range for LDH was 120.00 U/L to 230.00 U/L; the normal concentration range for AFU was < 40.00 U/L; and the normal concentration range for SA was 45.60 to 75.40 mg/dL. Before the prostate biopsy, plasma PSA levels and FIB concentrations were determined as well. To measure PSA and FIB levels, blood samples (about 3.50 mL) were collected. Samples were collected in plastic tubes with coagulant/separating glue for PSA analysis and in plastic tubes containing 3.80% sodium citrate for FIB detection. The normal concentration range for FIB was 2.00 to 4.00 g/L; the normal concentration range for PSA was < 4.00 ng/mL.

### 2.4 Statistical analysis

In total, 1202 cases of PCa were enrolled. Based on frequency analysis, AKP, LDH, AFU, FIB, and PSA did not have a normal distribution, so medians (percentiles) were used instead. To determine whether SA followed a normal distribution, the Kolmogorov-Smirnov test was employed on the means and standard deviations (SDs) in each group. Normally distributed data were analyzed using Student’s *t* test. Non-normally distributed data were analyzed using the Mann-Whitney U test or the Kruskal-Wallis H test. The correlation between two parameters was analyzed using the Spearman method. Categorical variables are presented as numbers (percentages), and the Chi-square test was used to compare the groups. The associations of different parameters with the bone metastasis status and HVD were evaluated by univariate and multivariate logistic regression analysis. To establish SA cut-off values, a receiver operating characteristic (ROC) curve was plotted and the resulting area under the curve (AUC) was calculated. The value with the largest Youden index (calculated as (sensitivity + specificity) + 1) was defined as the optimal cut-off value. Differences were considered to be statistically significant if *P* < 0.05. Statistical analysis was performed using Statistical Package for Social Sciences (SPSS) version 19.0 (SPSS, Inc., Chicago, IL, USA). The dynamic nomogram is introduced as a model, wherein the summation of individual patient scores on predictive factors enables the computation of their total score and subsequent determination of their risk for the outcome event. Variables exhibiting p values of 0.05 or lower in the logistic regression analysis were incorporated into the nomogram. Calibration curves and receiver operating characteristic (ROC) analysis were utilized to assess the predictive capabilities of the model. Additionally, decision curve analysis (DCA) was employed to evaluate the net benefits of clinical interventions guided by the model (R vision 4.1.3).

## 3 Results

### 3.1 Clinical characteristics of PCa patients

The characteristics of the patients are presented in **Table 1**. The location and quantity of bone metastases in PCa patients were assessed through ECT scanning as reported previously (32). The total number of patients diagnosed with bone metastases was 64.

**Table 1:** Clinical characteristics of selected PCa patients (*n* = 461).

Parameter	Values
Age (years)	70.00 (64.00, 76.00)
AKP (U/L)	73.00 (59.50, 95.00)
LDH (U/L)	194.00 (174.00, 224.00)
AFU (U/L)	16.00 (13.00, 20.00)
FIB (g/L)	3.13 (2.66, 3.65)
PSA (ng/mL)	34.87 (11.78, 125.30)
SA (mg/dL)	55.90 (50.75, 63.50)
Pathological grade	
Low (GS ≤ 7)	197 (42.73)
High (GS > 7)	242 (52.49)
Missing information	22 (4.77)
Pathological stage	
pT2	340 (73.75)
pT3	5 (1.08)
pT4	98 (21.26)
biopsy	18 (3.91)
ECT (bone metastasis burden)	
Without bone metastases	160 (34.71)
LVD patients	13 (2.82)
HVD patients	51 (11.06)
Suspicion	88 (19.09)
ECT not performed	149 (32.32)

Data are presented as median (P25, P75) or *n* (%). AKP, alkaline phosphatase; LDH, lactate dehydrogenase; AFU, α-L-fucosidase; FIB, fibrinogen; PSA, prostate-specific antigen; SA, serum sialic acid; PCa, prostate cancer; T, tumor; pathological grades are categorized as high grade and low grade using GS; pathological stage is assessed based on postoperative pathology results (not biopsy) in accordance with the 2021 TNM classification system.

### 3.2 The relationship between serum sialic acid and clinical characteristics

**Figure 2:**
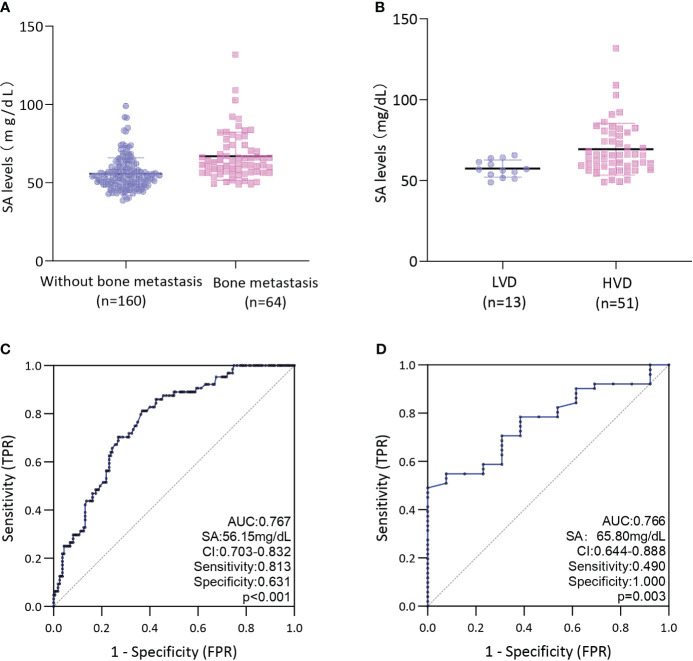
**(A)** Comparison of SA levels between bone metastases and without bone metastases in PCa patients; **(B)** Comparison of SA levels between LVD and HVD in PCa patients with bone metastases; **(C)** ROC curves for determination of cut-off value of SA levels regarding prediction of bone metastases in PCa patients; **(D)** ROC curves for determination of cut-off value of SA levels regarding prediction of HVD in PCa patients; HVD, high volume disease; LVD, low volume disease.

The median SA level in the cohort with bone metastases was found to be 62.70 mg/dL, which was observed to be significantly higher than in the non-bone metastasis cohort (53.75 mg/dl) (*P* < 0.001) (**Figure 2A**). Further examination of the HVD and LVD groups indicated that patients with HVD exhibited elevated SA levels in comparison to patients with LVD (65.70 mg/dL) (*P* = 0.003) (**Figure 2B**).

ROC curve analysis was conducted to determine the predictive value of pretreatment SA levels for bone metastases. As depicted in **Figure 2C**, a cut-off level of 56.15 mg/dL SA was obtained, with SA levels above this threshold being associated with a higher risk of bone metastases (AUC = 0.767; 95% CI: 0.703-0.832; *P* < 0.001; sensitivity: 0.813, specificity:0.631). Our assessment of the ROC curve derived from SA levels of patients with LVD or HVD yielded an AUC of 0.766 (95% CI: 0.644-0.888), with an optimal threshold value of 65.80 mg/dL (sensitivity: 0.490, specificity:1.000) (**Figure 2D**).

As shown in **Table 2**, subjects in the present study were also divided into two groups based on SA levels (≤ 56.15 mg/dL vs. > 56.15 mg/dL). A significant association was found between pretreatment SA levels and AKP, LDH, FIB, and PSA levels, Gleason score, pT stage, and bone metastasis (all *P* < 0.050). Moreover, we divided the databases into two categories based on their SA levels (≤ 65.80 mg/dL vs. > 65.80 mg/dL) (**Table 3**). In addition to AKP, LDH, FIB, and PSA levels, Gleason score, pT stage, and HVD, pretreatment SA levels were significantly associated with these parameters (all *P* < 0.050).

**Table 2:** Comparison of clinical parameters between groups presenting distinct ranges of SA levels (≤56.15 mg/dL vs. >56.15 mg/dL).

Parameter	SA ≤ 56.15 mg/dL (*n* = 237)	SA > 56.15 mg/dL (*n* = 224)	Statistics	*P*
Age (years)	70.00 (64.00, 75.50)	70.00 (64.00, 76.00)	−0.045	0.964*
AKP (U/L)	64.00 (53.00, 77.50)	82.00 (68.25, 118.50)	−9.386	0.000*
LDH (U/L)	187.00 (169.00, 210.00)	198.00 (179.00, 241.50)	−4.519	0.000*
AFU (U/L)	16.00 (13.00, 20.00)	16.00 (13.00, 20.75)	−0.870	0.384*
FIB (g/L)	2.76 (2.53, 3.09)	3.61 (3.17, 4.27)	−12.556	0.000*
PSA (ng/mL)	24.27 (10.50, 66.48)	66.67 (16.19, 216.95)	−5.239	0.000*
Gleason score (≤7 vs. >7)	115/107	82/135	8.712	0.003^#^
pT stage (T1-2 vs. T3-4)	197/31	143/72	24.536	0.000^#^
Metastatic patients	12 (10.60)	52 (46.80)	36.010	0.000^#^

Data are presented as median (P25, P75), *n/n*, or *n* (%). *Z-values; ^#^x2 values; AKP, alkaline phosphatase; LDH, lactate dehydrogenase; AFU: α-L-fucosidase; FIB, fibrinogen; PSA, prostate-specific antigen; SA, serum sialic acid; PCa, prostate cancer; T, tumor; pathological grades are categorized as high grade and low grade using GS; *P* < 0.05 is considered as statistically significant.

**Table 3:** Comparison of clinical parameters between groups presenting distinct ranges of SA levels (≤65.80 mg/dL vs. >65.80 mg/dL).

Parameter	SA ≤ 65.80 mg/dL (*n* = 365)	SA > 65.80 mg/dL (*n* = 96)	Statistics	*P*
Age (years)	71.00 (64.00, 76.00)	69.00 (61.25, 76.00)	−1.453	0.146*
AKP (U/L)	68.00 (57.00, 83.50)	102.00 (73.25, 152.50)	−7.556	0.000*
LDH (U/L)	191.00 (171.00, 214.00)	214.00 (181.25, 263.00)	−4.419	0.000*
AFU (U/L)	16.00 (13.00, 20.00)	16.00 (13.00, 19.75)	−0.392	0.695*
FIB (g/L)	2.94 (2.61, 3.34)	4.25 (3.64, 5.20)	−12.298	0.000*
PSA (ng/mL)	29.07 (10.85, 94.13)	94.12 (21.93, 344.28)	−5.350	0.000*
Gleason score (≤7 vs. >7)	166/182	31/60	5.422	0.020^#^
pT stage (T1-2 vs. T3-4)	286/67	54/36	17.757	0.000^#^
HVD patients	26 (66.70)	25 (100.00)	10.458	0.001^#^

Data are presented as median (P25, P75), n/n, or n (%). *Z-values; ^#^x^2^ values; HVD, high-volume disease; AKP, alkaline phosphatase; LDH, lactate dehydrogenase; AFU, α-L-fucosidase; FIB, fibrinogen; PSA, prostate-specific antigen; SA, serum sialic acid; PCa, prostate cancer; T, tumor; pathological grades are categorized as high grade and low grade using GS; P < 0.05 is considered as statistically significant.

### 3.3 Comparison of serum sialic acid levels between PCa patients with and without bone metastasis

Data were missing for three PCa patients without bone metastases. To determine the parameters that differ between the three groups, we excluded patients with missing data. The first group had no bone metastasis, the second group had LVD, and the third group had HVD. *P*1 denotes the outcome of comparing the group without bone metastasis to the LVD group, whereas *P*2 represents the result of comparing the group without bone metastasis to the HVD group. Lastly, *P*3 signifies the outcome of comparing the LVD group to the HVD group. The three groups are compared in **Table 4**. Between any two groups, there was no statistically significant difference in age and AFU levels. In addition, there were statistically significant differences in PSA and AKP levels between any two groups. The HVD group had higher FIB levels compared with the non-metastatic and LVD groups (*P* < 0.050). The HVD group had higher Gleason scores compared with the non-metastatic (*P* = 0.000) and LVD groups (*P* = 0.001). There were no statistically significant differences in FIB levels (*P* = 0.417), and Gleason scores (*P* = 0.340) between the group without metastatic disease and LVD patients. When the LVD group was compared to non-metastatic patients (*P* = 0.143) and the HVD group (*P* = 0.409), there was no statistically significant difference in LDH levels. There was, however, a statistically significant difference in LDH levels between the patients without metastatic disease and the HVD group (*P* = 0.000).

**Table 4:** Comparison of clinical parameters among non-metastatic (Group 1), LVD (Group 2), and HVD group (Group 3).

Parameter	Group 1 (*n* = 160)	Group 2 (*n* = 13)	Group 3 (*n* = 51)	*P*1 (sta)	*P*2 (sta)	*P*3 (sta)
Age (years)	71.00 (64.00, 75.00)	69.00 (68.00, 75.00)	68.00 (63.00, 76.00)	0.804 (−0.248*)	0.409 (−1.066*)	0.531 (−0.627*)
AKP (U/L)	67.50 (56.00, 78.00)	90.00 (69.00, 96.00)	122.00 (95.00, 276.00)	0.007 (−2.719*)	0.000 (−8.090*)	0.002 (−3.163*)
LDH (U/L)	185.50 (168.00, 211.50)	197.00 (178.00, 222.50)	212.00 (182.00, 273.00)	0.143 (−1.466*)	0.000 (−4.126*)	0.409 (−0.826*)
AFU (U/L)	16.00 (13.00, 20.75)	18.00 (15.50, 27.00)	18.00 (13.00, 23.00)	0.056 (−1.909*)	0.141 (−1.471*)	0.332 (−0.970*)
FIB (g/L)	2.96 (2.62, 3.39)	2.91 (2.75, 3.51)	3.63 (3.01, 4.47)	0.417 (−0.812*)	0.000 (−4.879*)	0.026 (−2.228*)
PSA (ng/mL)	31.01 (11.54, 79.01)	58.23 (28.56, 138.17)	215.70 (104.50, 507.60)	0.092 (−1.687*)	0.000 (−7.090*)	0.002 (−3.062*)
GS (≤7 vs. >7)	82/75	5/8	12/39	0.340 (0.911^#^)	0.000 (12.801^#^)	0.001 (10.458^#^)
SA > 56.15 mg/dL	59.00 (36.88)	8.00 (61.54)	44.00 (86.27)	0.079 (3.082^#^)	0.000 (37.771^#^)	0.101 (2.695^#^)
SA > 65.80 mg/dL	21.00 (13.13)	0.00 (0.00)	25.00 (49.02)	0.341 (0.906^#^)	0.000 (29.227^#^)	0.001 (10.458^#^)

Data are presented as median (P25, P75), n/n, or n (%). *Z-values; ^#^x^2^ values; LVD, low-volume disease; HVD, high-volume disease; P1, Group 1 vs. Group 2; P2, Group 1 vs. Group 3; P3, Group 2 vs. Group 3; sta, statistics; PSA, prostate-specific antigen; T, tumor; P < 0.05 is considered as statistically significant.

### 3.4 The relationship between serum sialic acid levels and HVD

To further examine the clinical effects of pretreatment SA levels in the diagnosis of bone metastases and HVD, a logistic regression analysis was conducted. The final model selection was made using a backward stepdown selection process. As shown in **Table 5**, elevated SA levels significantly predicted poor diagnostic outcomes of bone metastases in the univariate and multivariate analysis (P < 0.050). In addition, elevated AKP levels, high PSA levels, and high Gleason score were significantly associated with the diagnosis of bone metastases based on univariate and multivariate analysis (AKP: univariate analysis: HR = 1.034, P = 0.000 and multivariate analysis: HR = 1.029, P = 0.000; PSA: univariate analysis: HR = 1.007, P = 0.000 and multivariate analysis: HR = 1.005, P = 0.000; Gleason score: univariate analysis: HR = 3.023, P = 0.001 and multivariate analysis: HR = 4.372, P = 0.003) (**Table 5**). Interestingly, in HVD patients, elevated SA levels were significantly predicted. Both univariate and multivariate logistic regressions showed that elevated SA levels, PSA levels and high Gleason score are independently associated with HVD patients (all P < 0.050) (**Table 6**). However, HVD was not significantly correlated with AKP based on univariate and multivariate logistic regressions (**Table 6**).

**Table 5:** Univariate and multivariate logistic regression analysis of selected parameters in PCa and its bone metastases.

Variable	Univariate regression			Multivariate regression		
	HR	95% CI of OR	*P*-value	HR	95% CI of OR	*P*-value
Age	0.982	0.947-1.018	0.322	/	/	/
LDH	1.014	1.007-1.022	0.000	/	/	/
AKP	1.034	1.021-1.047	0.000	1.029	1.015-1.044	0.000
AFU	1.042	0.997-1.088	0.066	1.067	1.005-1.132	0.033
FIB	1.409	1.092-1.818	0.008	/	/	/
PSA	1.007	1.004-1.009	0.000	1.005	1.002-1.008	0.000
SA (mg/dL): >56.15 vs. ≤56.15	7.418	3.665-15.014	0.000	2.966	1.203-7.315	0.018
Gleason score	3.023	1.599-5.715	0.001	4.372	1.637-11.673	0.003

PCa, prostate cancer; LDH, lactate dehydrogenase; AKP, alkaline phosphatase; FIB, fibrinogen; PSA, prostate-specific antigen; HR, hazard ratio; P < 0.05 is considered as statistically significant; 95% CI: 95% confidence interval.

**Table 6:** Univariate and multivariate logistic regression analysis of selected parameters in LVD patients and HVD patients.

Variable	Univariate regression			Multivariate regression		
	OR	95% CI of OR	*P*-value	OR	95% CI of OR	*P*-value
Age	0.973	0.901-1.051	0.493	/	/	/
LDH	1.002	0.996-1.008	0.511	/	/	/
AKP	1.018	1.000-1.036	0.046	/	/	/
AFU	0.937	0.857-1.025	0.157	/	/	/
FIB	2.660	1.026-6.891	0.044	/	/	/
PSA	1.008	1.001-1.015	0.021	1.013	1.001-1.025	0.028
SA (mg/dL): >65.80 vs. ≤65.80	1.131	1.026-1.247	0.013	1.194	1.001-1.423	0.048
Gleason score	2.031	0.558-7.388	0.282	31.217	1.985-490.831	0.014

HVD, high-volume disease; LDH, lactate dehydrogenase; AKP, alkaline phosphatase; FIB, fibrinogen; PSA, prostate-specific antigen; HR, hazard ratio; P < 0.05 is considered as statistically significant; 95% CI, 95% confidence interval.

### 3.5 Nomogram model of bone metastasis and HVD

**Figure 3:**
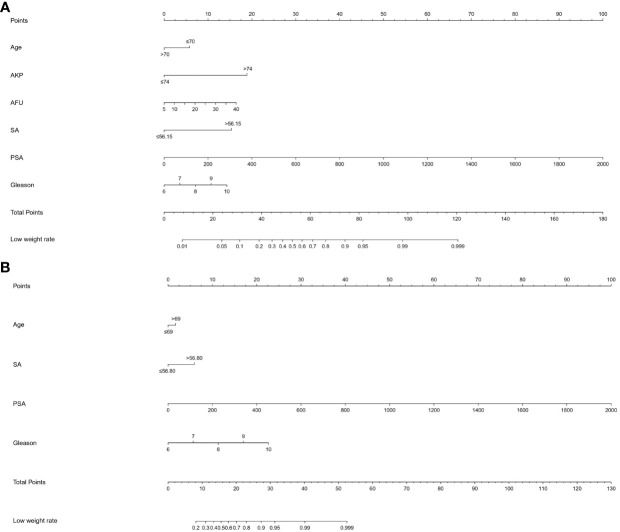
**(A)** A nomogram for predicting bone metastasis of PCa patients. **(B)** A nomogram for predicting HVD patients. The nomogram is used by summing all points identified on the scale for each variable. The total points projected on the bottom scales indicate the probabilities of bone metastasis and HVD. PCa, prostate cancer.

**Figure 4:**
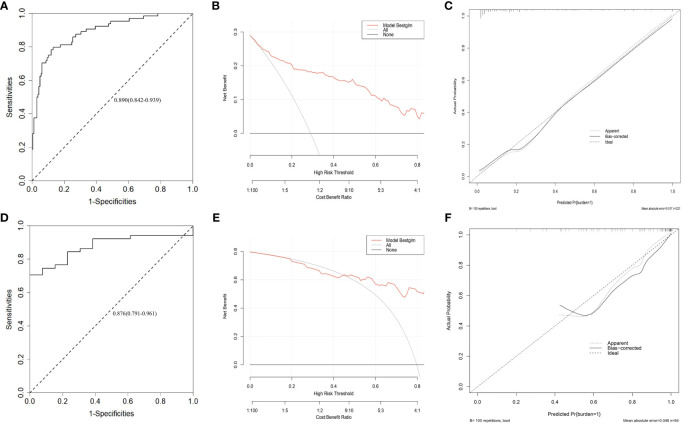
ROC curve **(A)**, DCA curve **(B)** and calibration curve **(C)** for assessing the discrimination and calibration of the nomogram in predicting the probabilities of bone metastasis. ROC curve **(D)**, DCA curve **(E)** and calibration curve **(F)** for assessing the discrimination and calibration of the nomogram in predicting the probabilities of HVD.

As shown in **Figure 3**, all significant factors for bone metastasis (**Figure 3A**) and HVD (**Figure 3B**) occurrence were integrated into the nomogram for predicting the probabilities of bone metastasis and HVD. Among them, age, SA levels, and AKP levels were divided according to their cut-off values. The ROC curve demonstrated good discrimination in predicting PCa (**Figure 4A**) and in predicting HVD (**Figure 4D**). In addition, the DCA curves and calibration curves indicated good agreement between the predicted and observed probabilities of bone metastasis (**Figures 4B, C**) and HVD (**Figures 4E, F**).

## 4 Discussion

PCa is a prevalent malignancy among men and is a significant contributor to mortality (33). PCa ranks as the third most frequently detected cancer in males, with an estimated 1.4 million global diagnoses projected for 2021, and it stands as the fifth primary contributor to cancer-related mortality. Various risk factors, encompassing genetic predisposition, advanced age, ethnic background, elevated testosterone levels, and lifestyle choices, significantly influence the initiation of PCa. Moreover, the incidence of PCa exhibits notable variation across diverse geographical regions, with developed and industrialized nations demonstrating a heightened prevalence (34, 35). Furthermore, research suggests that the incidence of PCa is positively correlated with advancing age among patients (34, 36). The overall incidence rate stands at 31 cases per 100,000 males across all age groups, while the lifetime cumulative risk is estimated at 3.9%. Notably, the prevalence of PCa among men aged 75 and above exceeds one in four individuals (37, 38). While the majority of cases are indolent and do not result in death, a substantial number of cases present with intermediate- or high-risk localized, locally advanced, or metastatic cancer despite treatment (39). The primary cause of PCa-related mortality is metastatic disease, which occurs when PCa cells proliferate beyond the prostate and disseminate to other organs, particularly the lungs, liver, bones, and lymph nodes (40). The likelihood of bone metastasis is significantly higher in patients with PCa who present with both osteoblastic and osteolytic lesions (41). The treatment of PCa has become increasingly diverse due to the wide range of available techniques (42). Local therapy is advantageous for many individuals, and patients who undergo radical prostatectomy have a better prognosis than those who receive radiation therapy (43). Presently, androgen deprivation therapy is the standard treatment for metastatic hormone-sensitive PCa. Furthermore, the combination of second-generation antiandrogens has been shown to increase the survival rate of men with metastatic castration-resistant PCa (M0 CRPC) (44, 45). However, aggressive treatment methods for PCa can result in significant adverse effects on continence and erectile function (46, 47). Therefore, it is imperative to accurately determine the clinical type and stage of PCa prior to initiating treatment.

Notably, clinically significant disparities between HVD and LVD have been observed in PCa, with LVD often being characterized as oligo-metastatic based on numerous studies (32, 48). Numerous clinical trials have established a correlation between (49–51). Furthermore, a consensus has yet to be reached on the definitions of HVD and LVD. Notably, both CHARTED and LATITUDE scores, which share many similarities, have been identified as significant predictors of survival. Consequently, we have opted to differentiate between HVD and LVD based on the CHARTED experiment’s definition (52).

Research has indicated that SA molecules are integral to the process of cancer metastasis. The progression of malignant tumors towards migration, invasion, and metastasis involves alterations in the levels of numerous intracellular and extracellular proteins, as well as modifications to normal cellular behavior, including extracellular matrix degradation, reduced cell adhesion, heightened cell motility, and augmented local microvessel formation, ultimately culminating in metastasis.

Prior research has demonstrated the significant involvement of adhesive glycoproteins in tumor metastasis, whereby the upregulation of 2-6-sialyltransferase and fibronectin expression influences the migratory capacity of mouse liver cancer cells (53). Furthermore, SA has been identified as a mediator of tumor cell adhesion to platelets, white blood cells, and vascular endothelial cells, while also reducing cell adhesion to collagen IV (a constituent of the basement membrane) and intercellular adhesion (54). Moreover, it has been demonstrated that the capacity to interact with nerve cell adhesion molecules can be augmented, thereby promoting the invasion, migration, and metastasis of neoplastic cells (55).

Despite the currently available antitumor pharmacological regimens, bone metastases originating from PCa have yet to be effectively managed. In comparison to antiangiogenic agents and matrix metalloproteinase inhibitors, pharmacotherapy targeting aberrant SA receptors may prove to be a more efficacious strategy in the future for combating tumor metastasis. AL10, a novel sialyltransferase inhibitor, has been shown to impede the adhesion, migration, actin expression, and invasion of malignant cells (56). Furthermore, SA, a molecule exhibiting a strong attraction towards anticancer drugs, presents a promising avenue for the advancement of antitumor therapy. Notably, highly metastatic tumor cells express certain adhesion molecules, including CD22 (57), and exhibit a heightened capacity for binding with SA. Consequently, anticancer drugs bound to SA can accumulate on the surface of tumor or metastatic cells, thereby augmenting the efficacy of the drugs. The development of such drugs has emerged as a prominent area of research.

The present study investigated the levels of SA in 461 patients prior to treatment, in conjunction with other pertinent indices and clinical characteristics. Furthermore, ROC curves were generated to predict bone metastases and HVD in PCa patients based on preoperative SA levels. The optimal diagnostic cut-off value for discriminating between patients with and without bone metastases was determined to be 56.15 mg/dL, while the optimal diagnostic cut-off for distinguishing between LVD and HVD was 65.80 mg/dL. We also presented AUC, sensitivity, and specificity as measures of accuracy. Our findings indicate that an elevated level of SA exhibits high sensitivity and specificity in the diagnosis of bone metastases in patients with PCa. Notably, pretreatment SA levels were significantly higher in patients with HVD compared to non-metastatic patients and patients with LVD. Multivariate regression analysis revealed that SA is independently associated with HVD. Therefore, elevated SA levels may serve as a valuable diagnostic tool for identifying osteometastases and HVD in PCa patients.

In this study, an examination was conducted on pretreatment SA levels in a cohort of 461 patients, alongside other pertinent assay indices and clinical characteristics. Consistent with previous research by Goswami K (58) and Cong Zhang (26) et al., our findings indicate a significant elevation in SA levels among patients with bone metastasis compared to those without, as well as higher SA levels in patients with HVD compared to those with LVD. Subsequently, patients diagnosed with PCa were subsequently stratified based on age, AKP, LDH, FIB, and PSA levels. The findings revealed a strong association between SA levels and LDH, AKP, FIB, and PSA. These results align with the conclusions drawn by Crook MA et al. (59), as no statistically significant correlation was observed between SA levels and age. Furthermore, a notable correlation was observed between SA levels and pathological grade, whereby higher pathological grades were accompanied by elevated SA levels. The patients were categorized into three groups based on the results of ECT: those without bone metastases, those with LVD, and those with HVD. The levels of SA exhibited a gradual increase across the three groups, with SA levels being lowest in the group without bone metastases, followed by the LVD group, and highest in the HVD group. ROC curves were then generated to assess the predictive value of preoperative SA levels for bone metastases and HVD. The optimal diagnostic cut-off points, determined by the maximum Younden index (60), were found to be > 56.15 mg/dL for distinguishing between bone metastases and the absence of bone metastases, and > 65.80 mg/dL for distinguishing between HVD and LVD. The picture also displayed the AUC, sensitivity, and specificity values for determining accuracy. It was evident that elevated SA levels exhibited high sensitivity and specificity in diagnosing bone metastases and HVD in PCa patients. Furthermore, SA levels were included in both univariate and multivariate analyses, which revealed their significance in diagnosing bone metastases and HVD. Consequently, elevated SA levels hold promise in the diagnosis of bone metastases and HVD in PCa patients.

Notwithstanding, there exist several constraints that necessitate consideration in this study. First, a retrospective study was conducted in a single location, which may have led to a statistically significant degree of selection bias. Second, despite the recognition of ECT as the “golden standard” for identifying skeletal metastases, this technique may still yield an erroneous diagnosis for patients with PCa. Third, despite the implementation of rigorous enrollment criteria, it was not feasible to entirely eliminate conditions that could impact plasma SA levels, such as varicose veins in the lower extremities and atherosclerosis. Finally, certain HVD patients may have had undetected visceral metastases due to inadequate assessment. To validate these findings, it will be imperative to conduct multicenter prospective studies with larger sample sizes.

In summary, pretreatment plasma SA levels exhibit a positive correlation with the number of bone metastases and are independently linked to HVD. These results suggest that SA levels in the bloodstream may serve as a potential marker for more accurate diagnosis and treatment of advanced PCa.

## 5 Conclusion

The present study established a correlation between SA levels and the clinicopathological characteristics of patients with PCa and HVD. Elevated SA levels prior to surgery could serve as an indicator of increased malignancy risk and advanced cancer stages. The findings of this study suggested that SA levels could serve as a valuable screening and prognostic marker for PCa and HVD.

## Author contributions

JS: Data curation, Software, Writing – original draft. TT: Data curation, Writing – review & editing. NW: Software, Writing – original draft. XJ: Software, Writing – original draft. LQ: Writing – review & editing. HC: Methodology, Writing – review & editing. ZL: Methodology, Writing – review & editing. JL: Methodology, Writing – review & editing. LY: Data curation, Writing – review & editing. DL: Methodology, Writing – review & editing.
