# Corrigendum: Pretreatment level of serum sialic acid predicts both qualitative and quantitative bone metastases of prostate cancer

**DOI:** 10.3389/fendo.2024.1466189

**Published:** 2024-08-26

**Authors:** Jingtao Sun, Tian Tian, Naiqiang Wang, Xuehui Jing, Laiyuan Qiu, Haochen Cui, Zhao Liu, Jikai Liu, Lei Yan, Dawei Li

**Affiliations:** ^1^ Department of Urology, Qilu Hospital of Shandong University, Jinan, China; ^2^ Respiratory and Critical Care Medicine Department, Qilu Hospital of Shandong University, Jinan, China; ^3^ Department of Urology, Yucheng People’s Hospital, Dezhou, China

**Keywords:** serum sialic acid, bone metastases, prostate cancer, low-volume disease, high-volume disease

In the published article, there was an error in the article type. Instead of “Review”, it should be “Original Research”.

The authors apologize for this error and state that this does not change the scientific conclusions of the article in any way. The original article has been updated.

